# *Trichoderma asperelloides* ethanolic extracts efficiently inhibit *Staphylococcus* growth and biofilm formation

**DOI:** 10.1371/journal.pone.0202828

**Published:** 2018-08-24

**Authors:** Simone S. Santos, Danillo G. Augusto, Patrícia A. Casaes Alves, Julia S. Pereira, Larissa M. B. Duarte, Poliana C. Melo, Eduardo Gross, Carla M. Kaneto, Aline Silva, Jane L. Santos

**Affiliations:** 1 Laboratório de Imunobiologia, Departamento de Ciências Biológicas, Universidade Estadual de Santa Cruz, Ilhéus, Brazil; 2 Laboratório de Genética Molecular Humana, Universidade Federal do Paraná, Curitiba, Brazil; 3 Centro de Microscopia Eletrônica, Departamento de Ciências Biológicas, Universidade Estadual de Santa Cruz, Ilhéus, Brazil; 4 Centro Federal de Educação Tecnológica de Minas Gerais, Departamento de Engenharia de Materiais, Belo Horizonte, Brazil; 5 Hospital Veterinário Departamento de Ciências Agrárias, Universidade Estadual de Santa Cruz, Ilhéus, Brazil; 6 Laboratório de Microbiologia, Departamento de Ciências Biológicas, Universidade Estadual de Santa Cruz, Ilhéus, Brazil; Tallinn University of Technology, ESTONIA

## Abstract

Fungi from the widely distributed genus *Trichoderma* are of great biotechnological interest, being currently used in a vast range of applications. Here, we report that high-molecular weight fraction (HWF) derived from *Trichoderma asperelloides* ethanolic extract exhibits antibiotic activity against staphylococcal biofilms. The antibacterial and anti-biofilm properties of *T*. *asperelloides* extracts were evaluated by well-established assays in *Staphylococcus aureus* ATCC strains (29213 and 6538) and in one clinical isolate from bovine mastitis. The HWF from *T*. *asperelloides* eradicated *S*. *aureus* by causing substantial matrix de-structuring and biomass reduction (*p* < 10^−5^) at concentrations as low as 2.3 μg mL^-1^. Additionally, we present ultra-structure analysis by the use of scanning electron microscopy as well as transmission microscopy, which showed that *T*. *asperelloides* killed cells through cell wall and membrane disturbance. Remarkably, the HWF from *T*. *asperelloides* killed *S*. *aureus* and eradicated its biofilms in a greater performance than gentamicin (*p* < 10^−5^), a known potent antibiotic against *S*. *aureus*. Our results indicate that extract from *T*. *asperelloides* may represent a promising candidate for the development of new antibiotics against gram-positive bacteria.

## Introduction

The genus *Trichoderma* comprises filamentous fungi species that are decomposers and components of the microflora, present in all soil types as well as the rhizosphere and organic matter [1,2]. *Trichoderma* exhibits efficient colonization ability as well as effect in plant health by controlling several phytopathogens [3].

One of the factors that contribute to the success full colonization of *Trichoderma* species is their ability of secreting enzymes and antibiotics [4]. The antimicrobial properties and metabolic versatility of *Trichoderma* fungi make them good candidates for drug discovery against infections caused by fungal, viral and bacterial pathogens, which cause diseases in plants and animals, including humans [5,6].

The *Trichoderma* anti-staphylococcal activity has been recently demonstrated [7]. *Staphylococcus aureus* is one of the major opportunistic pathogens that colonize a considerable portion of the human population [8,9,10]. *Staphylococcus* is responsible for causing several diseases, which varies from skin infections to bacteremia, being associated with high rates of morbidity and mortality [11]. An important factor that affects its pathogenicity is its ability to firmly adhere to prosthetic materials and the formation of biofilms on several surfaces, which includes pacemakers, implants and catheters. Additionally, *Staphylococcus* produces a variety of toxins, besides their ability to develop resistance against antimicrobial agents [12,13].

The resistance of *S*. *aureus* against antimicrobial agents, along with its ability to form biofilms, imposes serious difficulties in treating their infections [14]. Since the first use of penicillin, this species has been shown a notable ability to quickly adapt and acquire resistance to newly launched drugs [15]. The biological activities of *Trichoderma* species on controlling *S*. *aureus* and other pathogens make them promising anti-bacterial candidates for further investigation [16]. Here, we demonstrated that ethanolic extracts and derivate fractions from *T*. *asperelloides* efficiently destroyed biofilms produced by *S*. *aureus* ATCC strains and one clinical isolate.

## Methods

### *T*. *asperelloides* culture and ethanolic extract preparation

*T*. *asperelloides*, obtained from the Agro industrial Laboratory and Applied Microbiology (Universidade Estadual de Santa Cruz, Ilhéus-Ba, Brazil), was previously identified by morphological and molecular markers [17]. A total of 1×10^8^ spores were grown in 90 mm Petri dishes (n = 40 plaques) containing 20 mL of dextrose potato agar (PDA), kept at room temperature for 10 days until sporulation. Subsequently, all spores and mycelium were collected from the plates using 5 mL of ethanol (99.5% PA) for each plate. Subsequently, ethanolic extract (TE) was incubated under gentle stirring conditions in a TE143® homogenizer for 24 hours at room temperature. After homogenization, the entire extract was dried in SPEEDVARS AG 22331 (Hamburg Concentrate 5301) and ressuspended in 10 mL of phosphate buffered saline (PBS), pH 7.4. The amount of 5 mL was used for quantification and the remaining 5 mL were subjected to fractionation. The high molecular weight fractions (HWF, 1mL) and low molecular weight fractions (LWF, 4mL) derived from the *T*. *asperelloides* ethanolic extract were separated by centrifugation using a Centricon membrane (10kDa pore) 2370g. Protein quantification of total extract (TE), high molecular weight (HWF) and low molecular weight (LWF) fractions were performed by Qubit Fluorometric Quantitation (ThermoFisher).

### Bacterial strains

We analyzed two biofilm-forming *S*. *aureus* strains (ATCC 29213 and ATCC 6538) and a non-forming-biofilm strain *S*. *epidermidis* (ATCC 12228) that were kindly provided by Fundação Oswaldo Cruz, FIOCRUZ, Rio de Janeiro, Brazil). We also analyzed a clinical isolate strain of *S*. *aureus* from bovine mastitis, identified as strain 184 [18].

### Antimicrobial test through growth curve

*S*. *aureus* strains were stored in brain heart infusion medium (BHI) and incubated for 24 hours at 37°C for activation. The bacterial concentration was adjusted to 10^8^ colony-forming units per milliliter (CFU mL^-1^). After this period, the strains were cultivated in 96-well polystyrene plates and treated three different *T*. *asperelloides* ethanolic extracts: total extract (TE), molecular weight fractions (HWF) and low molecular weight fraction (LWF) at four different concentrations– 4.6, 2.3, 1.1 and 0.5 μg mL^-1^. As a positive growth control, we used untreated bacteria (PBS only) and a negative growth control, bacteria treated with gentamicin at 4.6 μg mL^-1^. The total volume was 200 mL in each well. The OD_600_ (600nm) was analyzed at different times (2, 4, 6, 8, 24 and 48 hours).

### Growth conditions and anti-biofilm activity evaluation

To evaluate the biofilm reduction due to *T*. *asperelloides* extracts, we used a method previously described by Leite et al., [19] with adaptations. Since we aimed to observe biofilm formation, we cultured onto 96-well polystyrene plates with BHI medium, in triplicates, until it reached the optical density of 0.5 at 600nm per well, which corresponds to approximately 10^8^ CFUmL^-1^. Next, we cultured the plates for 24 hours at 37°C to observe biofilm growth. Supernatant was then discarded and 100 μL of BHI was added in the wells with biofilm. Wells with biofilm and BHI medium were treated with increasing concentrations of total ethanolic extract (TE), high-molecular-weight fractions (HWF) and low-molecular-weight fractions (LWF): 0.5 μg mL^-1^, 1.1 μg mL^-1^, 2.3 μg mL^-1^ and 4.6 μg mL^-1^. Gentamicin (GIBCO-Invitrogen) at 4.6 μg mL^-1^ was used for comparisons. Both gentamicin and *T*. *asperelloides* fractions were initially diluted in PBS (total volume 100 μL) and then mixed with BHI medium containing biofilm. As control, we used the biofilm treated only with PBS. The plates with biofilm treated with gentamicin, *T*. *asperelloides* fractions and controls were incubated at 37°C without agitation for 24 hours. The supernatant was then discarded and 200 μL of NaCl 0.9% was added to suspend the biofilm. This suspension was divided in two equal parts: 100 μL was used for absorbance OD_600_ analysis; the other part was diluted (1:10) and 15ul, in triplicate, were transferred to Petri dishes with trypticsoy agar (TSA). Finally, the plates were incubated at 37° C for 24 hours.

### Biofilm biomass quantification

We evaluated the biofilm biomass production by the crystal violet method as described in [17]. After culturing for 24 hours, we treated with TE, HWF and LWF at concentration of 2.3 μg mL-1. After 24 hours of treatment, the plate wells were washed with saline solution (0.9% NaCl) and fixed with 200 μL of methanol, which was removed after 15 minutes. Then, the biofilm was allowed to dry at room temperature before adding 200 μL of crystal violet (1%v/v). After 5 minutes, the biofilm was gently washed with distilled water. Subsequently, the biofilm biomass was photo documented with an optical microscope (40x) and 100 μL of acetic acid (33% v/v) was added to each well (to release and dissolve the stain). The absorbance of the resulting solution was evaluated in triplicate in a microplate reader at 570nm by (Molecular Devices, Sunnyvale, California EUA).

#### Minimum inhibitory concentration of HWF in planktonic cultures

The HWF activity was tested in three *S*. *aureus* strains (ATCC 29213, ATCC 6538 and clinical isolate 184) and *S*. *epidermidis* (ATCC 12228) by double dilution method, according to the Clinical Institute and Laboratory Standard (CLSI) guidelines [20].

### Electronic microscopy

After observing biomass reduction, the effect of HWF on cell morphology was evaluated by scanning electron microscopy (SEM) and transmission electron microscopy (TEM). For SEM, the cells were properly activated for 24 hours and 10^8^ cells were transferred to wells with glass coverslip at the bottom and BHI medium. The cells were incubated for 24 hours at 37°C for biofilm formation and were subsequently treated with HWF at 2.3 μg mL^-1^, gentamicin (GIBCO, Invitrogen) at 2.3μg mL^-1^ and PBS only (control). Fixation was performed with 2.5% glutaraldehyde in a 0.1 M sodium cacodylate buffer at pH 7.2. After 24 hours, we washed three times with cacodylate buffer, 10 minutes each. The samples were dehydrated with 50%, 60%, 70%, 80%, 90% and 100% ethanol, dried on LEICA CPD300 equipment, gilded and photodocumented by scanning electron microscope Quanta 250 (FEI company). For ultra structural analysis, biofilm samples were formed in 2 mL tubes. Fixation and rinsing were performed as described for SEM. Cells were post-fixed in 1% osmium tetroxide, 0.8% potassium ferrocyanide (SIGMA) in 0.1 M sodium cacodylate buffer for 1 hour and rinsed in the same buffer (three times, 10 min each). After dehydration in an acetone series (30%, 50%, 70%, 90% and 100% for 10 min), the samples were included into the Epon resin. Sections with depth of 70 nm were obtained by ultramicrotomer (Leica EMUC 6, Vienna, Austria). These sections were collected in copper grids and contrasted with 5% aqueous solution of uranyl acetate (20 min) and 2% lead citrate (5 min). The observations and images were obtained with a Morgagni FEI 268D transmission electron microscope at 80 kV.

### Data analysis

All treatments have been performed in three independent experiments, each one of them with three replicates. Statistical descriptive and comparisons were performed using GraphPad Prism. Global differences among multiple treatments were evaluated using factorial ANOVA (analysis of variance) with post-hoc Tukey HSD (honestly significant difference); t-test was used for pairwise comparison. The significant *p*-value threshold was *p* = 0.05.

## Results

### Ethanolic extracts from *T*. *asperelloides* controlled bacterial growth

**Fig 1:**
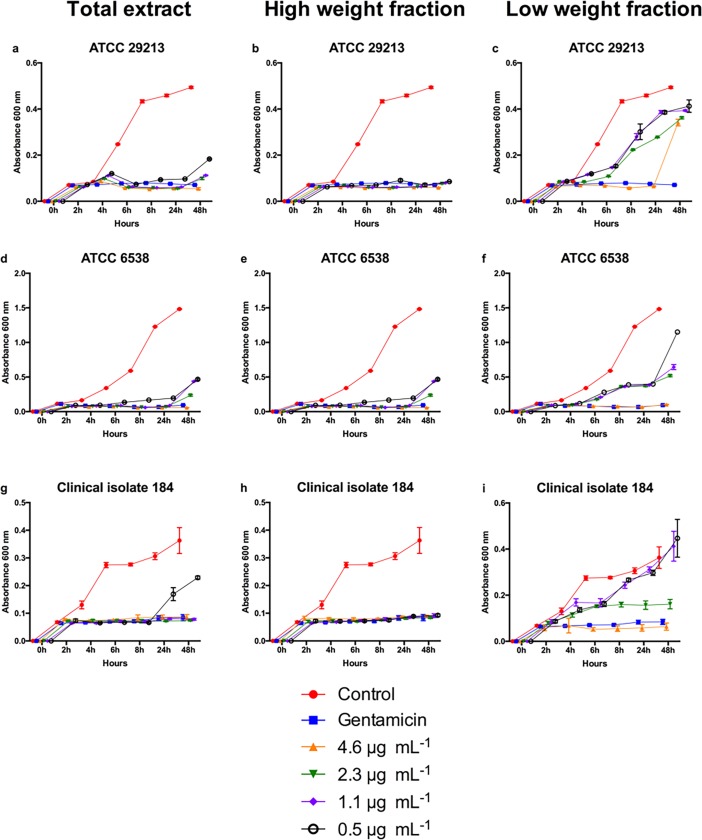
Extracts from *T*. *asperelloides* exhibit antibacterial activity. Bacterial growth curve of *S*. *aureus* ATCC 29213 (a, b and c), ATCC 6538 (d, e and f) and clinical isolate 184 (g, h and i), all treated in a period from 2, 4, 6, 8, 24 and 48 hours, with TE, HWF and LWF in 0.5 μg mL^-1^, 1.1 μg mL^-1^, 2.3 μg mL^-1^and 4.6 μg mL^-1^concentrations. n = 3, in triplicate. TE: total extract; HWF: high weight fraction; LWF: low weight fraction. For the clinical isolate, higher concentration (1.1 μg mL^-1^ or above) of TE was required to control bacteria growth comparably to gentamicin (Fig 1G). However, HWF was effective on controlling the clinical isolate growth even in low concentration (Fig 1G). Interestingly, the LWF of the ethanolic extract had a poor performance in controlling *S*. *aureus* (Fig 1C, 1F, and 1I).

By analyzing the growth curve of different *S*. *aureus* strains cultured with different concentrations of ethanolic extracts from *T*. *asperelloides*, we demonstrated that TE and HWF have strong effect on killing *S*. *aureus* (Fig 1). For both ATCC 29213 and ATCC 6538 strains, the effect of all TE and HWF tested concentrations showed similar effect to the observed for the antibiotic gentamicin (Fig 1A, 1B, 1D and 1E; *p* > 0.05).

### Ethanolic extracts from *T*. *asperelloides* exhibit anti-biofilm activity

**Fig 2:**
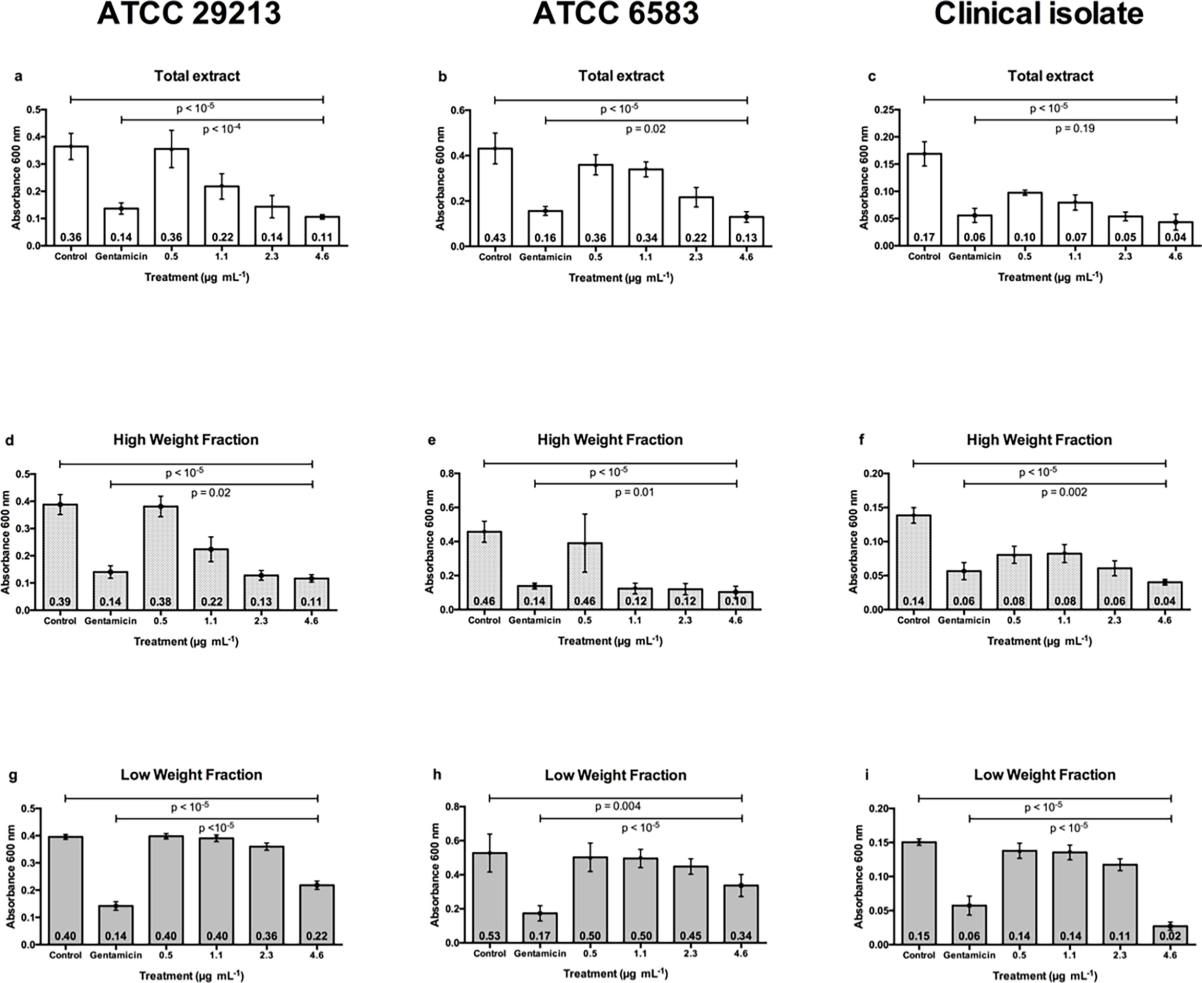
High weight fraction of *T*. *asperelloides* efficiently eradicates *S*. *aureus* biofilm. *Staphylococcus* biofilm growth was evaluated by absorbance in ATCC 29213 (a, d and g), ATCC 6538 (b, e and h) and clinical isolate 184 (c, f and i), after 24 hours in different concentrations. Control represents non-treated biofilm. The gentamicin-treated biofilm was at concentration of 4.6 μg mL^-1^. n = 3, in triplicate; numbers inside columns represent the mean and error bars represent standard deviation. TE: total extract; HWF: high weight fraction; LWF: low weight fraction.

**Fig 3:**
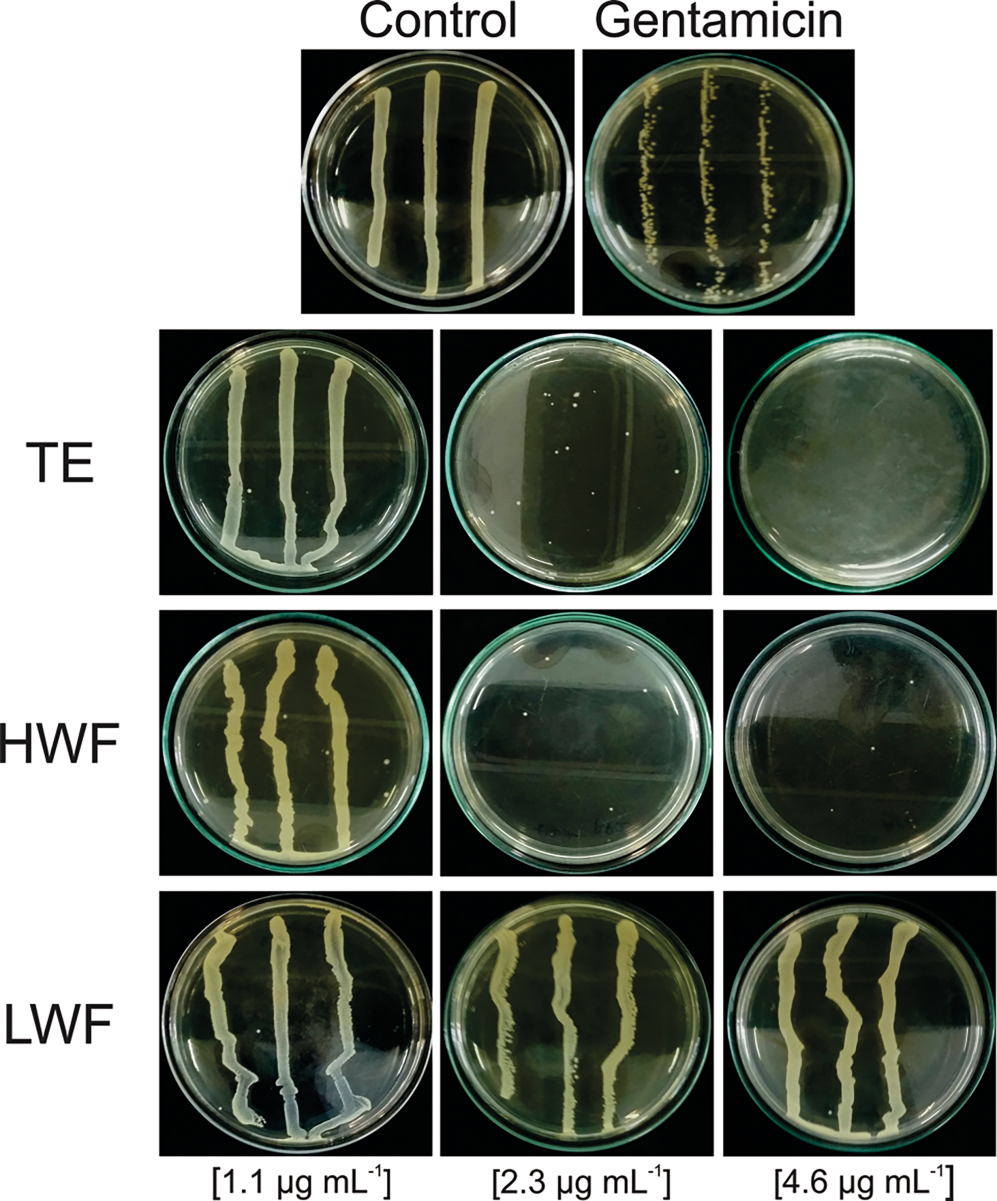
Qualitative evaluation of *T*. *asperelloides* antibiotic activity. Biofilm of *S*. *aureus* strain ATCC 29213 was treated for 24 hours *T*. *asperelloides* extracts. Dilutions 1:10 of this biofilm were ressuspended in 0.9% NaCl and were cultured in Petri dish containing trypticsoy agar medium for 24 hours. The control represents untreated biofilm. The biofilm treated with gentamicin was at 4.6 μg mL^-1^. TE: total extract; HWF: high weight fraction; LWF: low fraction of weight.

**Fig 4:**
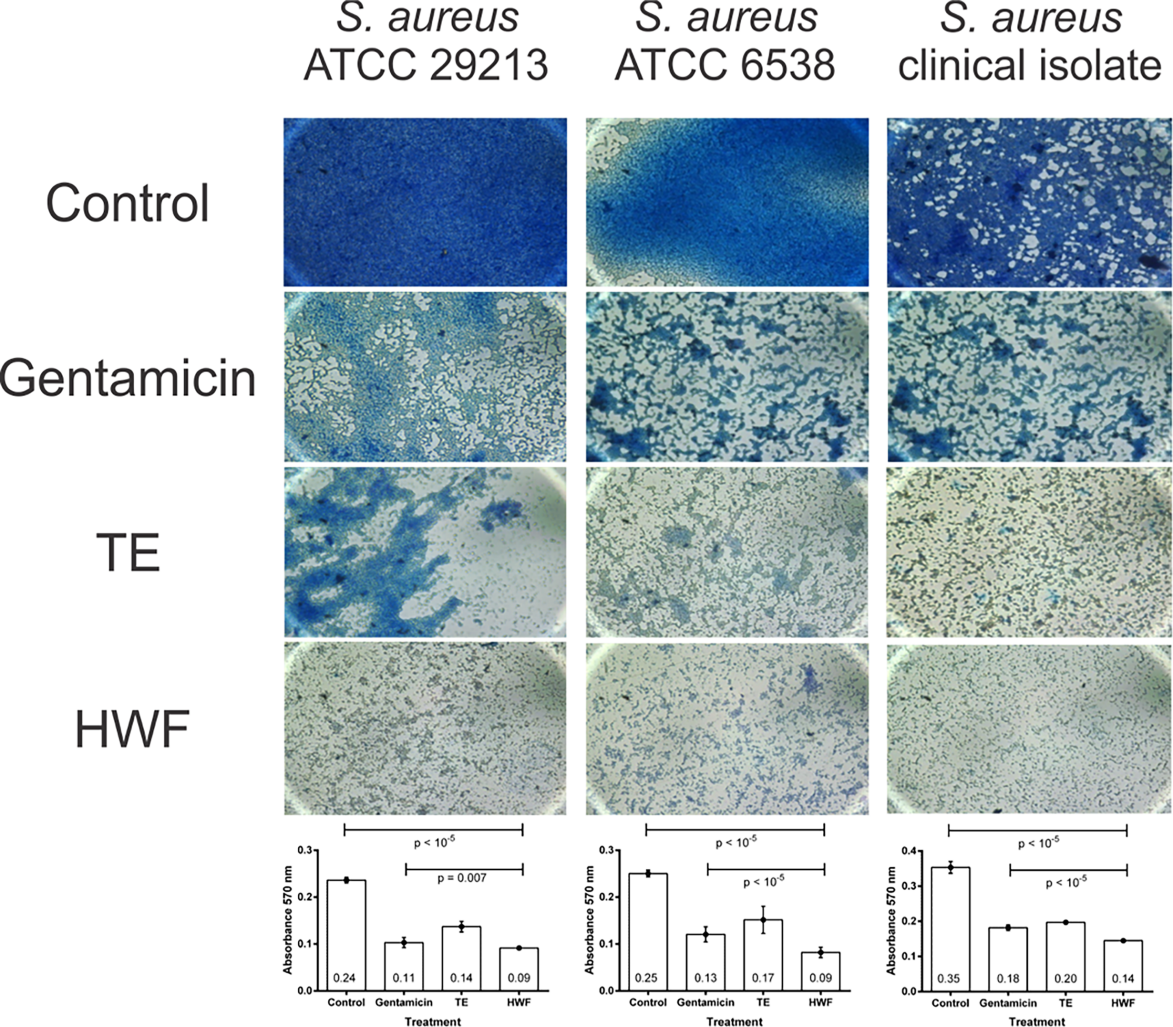
*Trichoderma* extracts reduce *S*. *aureus* biomass. Biomass was evaluated by crystal violet method. Microtiter plates were photographed under an optical microscope and quantified by a microplate reader at 570 nm. Biofilms formed after 24 hours were treated with TE and HWF at 2.3 μg mL^-1^. The control represents untreated biofilm. The biofilm treated with gentamicin was at 4.6 μg mL^-1^. n = 3, in triplicate; numbers within the columns represent the mean and error bars represent standard deviation. TE: total extract; HWF: high weight fraction.

After treating biofilm-forming strains with different concentrations of ethanolic extracts for 24 hours, we showed that TE and HWF (Fig 2A, 2B, 2C, 2D, 2E and 2F) efficiently controlled biofilm formation in comparison to controls (*p* < 10^−5^). We also observed that the anti-biofilm activity was dose-dependent, with a higher dosage having a stronger effect. Except the TE treatment in the clinical isolate (*p* = 0.19; Fig 1C), all experiments showed that a high dosage of TE and HWF of *T*. *asperelloides* had a more efficient effect on controlling biofilm than gentamicin (*p* < 0.02). A qualitative evaluation of the ethanolic extracts anti-biofilm activity in ATCC 29213 is shown in Fig 3. We observed complete inhibition of bacterial growth in TSA medium with TE and HWF at 4.6 μg mL ^-1^, different from which was observed for gentamicin (Fig 3). The quantitative and qualitative assessment of the anti-biofilm control observed for TE and HWF was also demonstrated by biomass quantification analysis. Using violet crystal technique, we observed biofilm biomass reduction, and the antibiofilm effect of HWF was observed at 2.3 μg mL ^-1^ (Fig 4).

Comparably to which was observed in bacterial growth analysis, the LWF was not consistently effective on controlling biofilm formation (Fig 2G, 2z and 2I). In these experiments, gentamicin had stronger effect than high concentrations (4.6μg mL^-1^) of LWF (*p*< 10^−5^; Fig 2G and 2H). For clinical isolate 184, LWF only efficiently controlled biofilm at high dosage (Fig 2I).

#### Minimal inhibitory concentration (MIC) of HWF for planktonic cultures

The observed MIC of HWF for all *S*. *aureus* strains was 4.6 μg mL^-1^, whereas the MIC for *S*. *epidermidis* was 9.2 μg mL^-1^.

### *Trichoderma* ethanolic extract damages the bacteria cell wall

**Fig 5:**
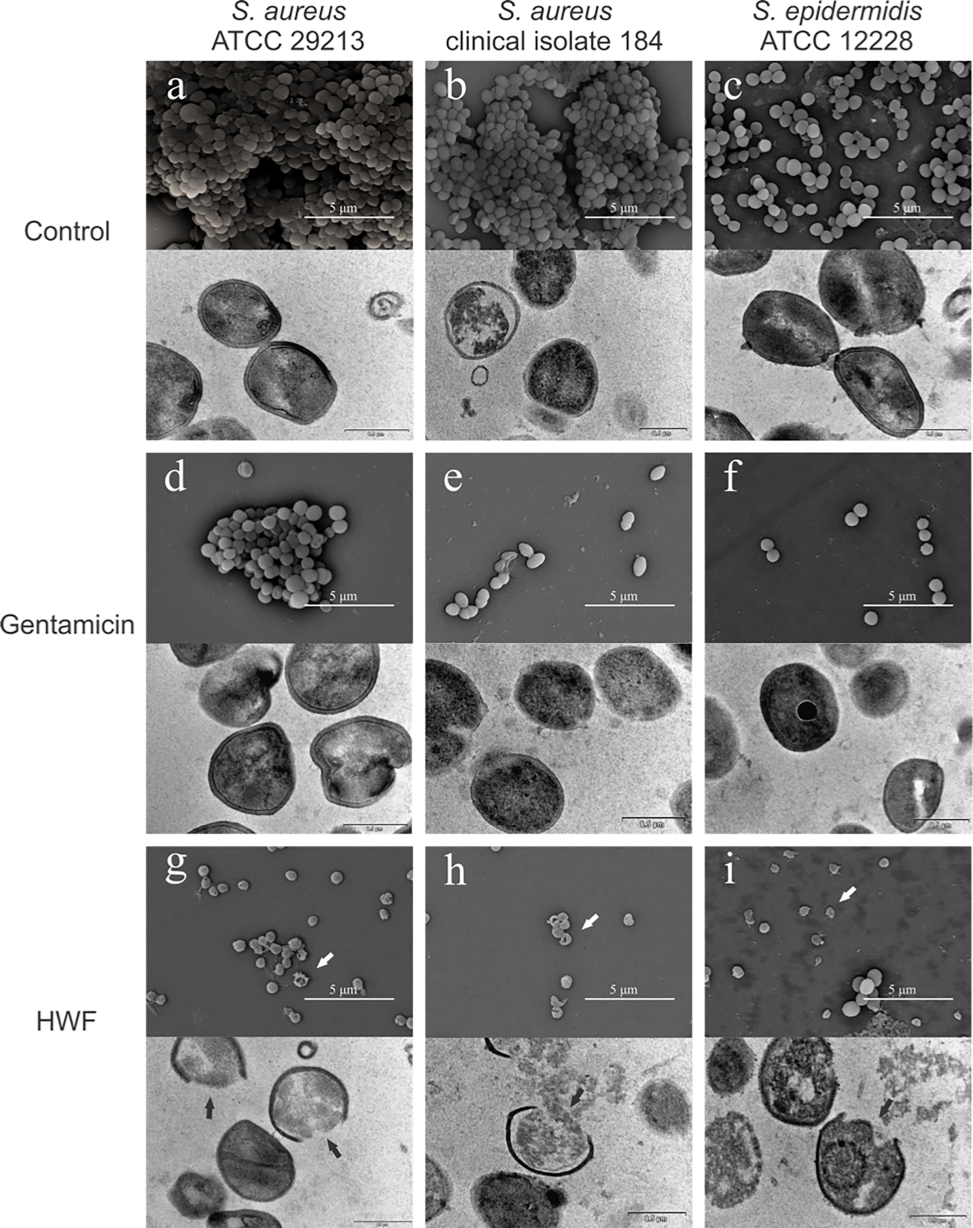
Extract from *Trichoderma* damages *S*. *aureus* cell wall. Effect of 24-hour treatment with high weight fraction of *T*. *asperelloides* on biofilms formed by *S*. *aureus* ATCC 29213, clinical isolate 184 and non-biofilm-forming *S*. *epidermidis* ATCC 12228. Scanning electron microscope images (top part of each image) and transmission electron microscope (bottom part). The images a, b and c were obtained from untreated biofilm (control); the images d, e and f were obtained from biofilm treated with gentamicin; and the images g, h and i were obtained from biofilm treated with *T*. *asperelloides* high weight fraction (HWF). The arrows indicate damaged cells.

Using electron microscopy, we observed that biofilm cells treated with HWF suffered deformation, volume reduction and cell wall degradation (Fig 5G, 5H and 5I). This was not observed in gentamicin treated cells (Fig 5D, 5E and 5F). We further analyzed planktonic form of the non-biofilm-forming staphylococcal strain (*S*. *epidermidis* ATCC 12228) to compare the effect of *Trichoderma* extracts in both biofilm forming and non-forming bacteria. *S*. *epidermidis* was also susceptible to TE and HWF treatments and suffered similar morphological structural damages compared to the sessile forms (Fig 5I). Electron microscopy images suggest loss of cell adhesion (Fig 5G, 5E, 5H).

## Discussion

The ability of certain fungi to produce bactericidal or bacteriostatic compounds has aroused much interest in their use for biotechnological and medical applications [21, 22, 23]. *T*. *asperelloides* is not as well-characterized as other species in this genus and has been incorrectly identified as *T*. *asperellum* in past [24]. Antibacterial effect was reported for *T*. *asperellum*, *T*. *viride*, *T*. *hamatum*, *T*. *koningii*, *T*. *atroviride*, *T*. *harzianum*, *T*. *longibrachiatum* and *T*. *citrinoviride*, being peptaibol one of the active compounds identified [7]. Apart from peptaibol, low molecular weight compounds have been identified in other studies that analyzed *Trichoderma* species, such as the trichorzins HA e MA isolated from *T*. *harzianum*, sesquiterpenes and cyclopeptide from *T*. *asperellum* [25,26,27].

Here, demonstrated that *T*. *asperelloides* extract controlled *S*. *aureus* growth more efficiently than gentamicin. Additionally, our experiments pointed out that the major antibacterial compounds have high molecular weight. Our study is the first to demonstrate the effect of the endophytic *T*. *asperelloides* on inhibiting biofilms of *S*. *aureus* ATCC and a mastitis clinically isolated strain. Similar to which we observed in cell growth experiments, only the high weight fraction was effective on controlling the biofilm formed by *S*. *aureus*. This observation points out that this fraction is the major candidate for identification of the anti-biofilm compounds produced by *Trichoderma*. Additionally, we showed that *T*. *asperelloides* extracts eliminated biofilms more efficiently than gentamicin.

The importance of our results is highlighted by the fact that the biofilm-forming capacity of *Staphylococcus* strains is one of its highly important characteristics in terms of adaptation [28]. Microorganisms that form biofilms have physiological modifications that usually allow them to become more resistant [29]. Without their biofilm protection, these bacteria would be more susceptible to host immunological defense or even to antibiotics [30].

In order to kill or remove biofilms, antimicrobials must penetrate the polysaccharide matrix to have access to the bacteria cells, which makes the removing of the biofilms a difficult and complex process [31]. As example, several studies have reported the inefficiency of peracetic acid and sodium hypochlorite to complete remove *S*. *aureus* biofilm cells from stainless steel and polypropylene surfaces [16, 32, 33, 34]. The efficient biofilm elimination promoted by *Trichoderma* extract was further demonstrated by the decreasing of biomass. Reducing *S*. *aureus* biomass could be a strategy to be explored to avoid biofilm formation or to eliminate formed biofilm on polystyrene or polypropylene surfaces.

The drastic reduction of the number of viable cells inside the biofilm, as well as the breaking of the cell wall observed in both SEM and TEM, suggest that the high molecular-weight compounds spread well into the biofilms and cause deficient cell adhesion. To evaluate if cell damage caused by *Trichoderma* extract was restricted to biofilms, we also treated *S*. *epidermidis* (Fig 5I). For microscopy, we treated with HWF at 2.3 μg mL^-1^, which was lower than the MIC (4.6 μg mL^-1^, for *S*. *aureus* and 9.2 μg mL^-1^ for *S*. *epidermidis*). The microscopy analysis of *S*. *epidermidis* revealed similar impact in a non-biofilm-forming strain. This evidences the possible broad application of *Trichoderma* extracts.

The biofilm formation is regulated by the expression of the intracellular polysaccharide adhesin (PIA), which mediates cell-to-cell adhesion [35]. A strategy to control biofilm formation, especially in dental plaque, has focused on the polysaccharides in its matrix. The (1 → 3), (1 → 6)-α-D-glucans are structurally and functionally essential biofilm constituents, therefor being good candidates for new studies. It has been shown the potential of the enzymes and (1 → 3) -α-glucanases for limiting biofilm formation [36, 37]. Additionally, Shiitake (*Lentinula edodes*) extracts that possess alpha glucanase degrade *Streptococcus mutans* glucans that are insoluble in water [38]. The identification of the high molecular compound produced by *T*. *asperelloides* that was able to control both *S*. *aureus* growth and biofilm formation would be beneficial to understand the mechanism behind its antibacterial activity and could open doors for drug discovery.

In addition to ATCC strains, we analyzed *S*. *aureus* clinical isolate from bovine mastitis. Consequently, our results suggest that high molecular-weight fractions from *Trichoderma* have promising biotechnological potential in veterinary medicine. *S*. *aureus* is a pathogen that challenges cattle farming by causing a variety of diseases, which includes skin infections, bacteremia, endocarditis, peritonitis, urinary tract infections, septicemia and others. Besides, *S*. *aureus* is an opportunistic pathogen frequently involved food poisoning due its capacity of forming biofilms on several surfaces, which causes a variety of contamination issues [8–11].

## Conclusions

Our findings corroborate previous studies that showed the existence of antibacterial properties in *Trichoderma* composites. We now demonstrated, for the first time, that high molecular weight compounds from *T*. *asperelloides* are the ones responsible for killing *S*. *aureus* and have drastic effect on reducing biofilm by disturbing the cell wall and membrane. We also demonstrated that the anti-biofilm control performed by *T*. *asperelloides* extract was more efficient than the one by gentamicin. Our study proves that molecular characterization of this fungal high molecular fraction as well as the comprehension of the mechanism behind anti-biofilm destruction could result in a broad range of biotechnological and medical applications.
